# Structural Characterization of Intrinsically Disordered Proteins by NMR Spectroscopy

**DOI:** 10.3390/molecules180910802

**Published:** 2013-09-04

**Authors:** Simone Kosol, Sara Contreras-Martos, Cesyen Cedeño, Peter Tompa

**Affiliations:** 1 VIB Department of Structural Biology, Vrije Universiteit Brussel, Brussels 1050, Belgium; E-Mails: sara.contreras.martos@gmail.com (S.C.M.); cesyen.cedeno@vub.ac.be (C.C.); 2 Institute of Enzymology, Biological Research Center, Hungarian Academy of Sciences, Budapest 1518, Hungary

**Keywords:** IDPs, NMR spectroscopy, in-cell NMR, ^13^C-direct detected NMR

## Abstract

Recent advances in NMR methodology and techniques allow the structural investigation of biomolecules of increasing size with atomic resolution. NMR spectroscopy is especially well-suited for the study of intrinsically disordered proteins (IDPs) and intrinsically disordered regions (IDRs) which are in general highly flexible and do not have a well-defined secondary or tertiary structure under functional conditions. In the last decade, the important role of IDPs in many essential cellular processes has become more evident as the lack of a stable tertiary structure of many protagonists in signal transduction, transcription regulation and cell-cycle regulation has been discovered. The growing demand for structural data of IDPs required the development and adaption of methods such as ^13^C-direct detected experiments, paramagnetic relaxation enhancements (PREs) or residual dipolar couplings (RDCs) for the study of ‘unstructured’ molecules in vitro and in-cell. The information obtained by NMR can be processed with novel computational tools to generate conformational ensembles that visualize the conformations IDPs sample under functional conditions. Here, we address NMR experiments and strategies that enable the generation of detailed structural models of IDPs.

## 1. Introduction

Intrinsically disordered proteins are involved in many essential biological processes and can be found in animals, plants, and to a lesser extent in bacteria and archaea [1]. Characteristically, intrinsically disordered proteins (IDPs) and intrinsically disordered regions (IDRs) lack stable secondary or tertiary structure and are highly flexible molecules. This results in conformational heterogeneity, and consequently, under physiological conditions, every IDP molecule samples a range of conformers over time. Some conformers of the same molecule might resemble a random coil structure, while others have structured elements called transient secondary structure [2]. 

The discovery and successive investigation of proteins that function without a well-defined structure challenged and changed the structure-function paradigm. More so, when it became clear, that for the so-called entropic chains, disorder is the one trait that enables the proteins to execute their function [2]. And although many IDPs undergo binding-induced folding [3], some stay significantly disordered when bound to another protein, resulting in the formation of heterogeneous, “fuzzy” complexes [4]. This structural flexibility offers certain advantages: a combination of low affinity and high specificity in the case of binding-induced folding IDPs, which is exploited in cell-signaling pathways, and a higher binding promiscuity, frequently utilized in hub proteins of large interaction networks [3,5]. Interestingly, the dynamic behavior of IDPs seems to be an evolutionary conserved feature, similar to structural conservation in folded proteins. A recent study showed conservation of backbone dynamics of homologues of the p53 transactivation domain, while amino acids and secondary structure propensities were not conserved [6].

Despite the lack of a stable secondary and tertiary structure, IDPs play important roles in critical, mostly regulatory, cellular functions such as differentiation, transcription regulation, DNA condensation, mRNA processing, and apoptosis [2,7]. These functions are frequently modulated by post-translational modification such as phosphorylation or glycosylation [8]. Numerous IDPs have been connected to diseases, examples include alpha-synuclein in Parkinson’s, tau protein in Alzheimer’s, or the role of tumor suppressor p53 in cancer formation [8]. The biological importance of IDPs becomes evident with the severity of the pathological conditions associated with malfunctions of IDPs. Due to their involvement in disease, IDPs became of interest for drug discovery, and strategies to create molecules that would inhibit disorder-based interactions of IDPs have been developed [5]. Depending on the pathogenesis, drugs have been designed to either inhibit binding, like in the case of the oncoprotein c-Myc [9], or to block amyloid aggregation, the key event in neurodegenerative disease [10]. But regardless of the desired molecular effect of the drug, atomic-scale structural characterization of the target allows rational drug design and the discovery of more effective treatments [5,10]. Therefore, the demand for structural and molecular dynamics data of IDPs is growing. Most experimental and analytical techniques, however, have been created for the investigation of structured proteins, necessitating the development of new experiments and techniques [11]. Additionally, adequate computational techniques had to be invented to interpret experimental data to obtain accurate structural models of IDPs. Algorithms such as flexible-meccano [12] have been created specifically for the generation of ensembles of conformations, the form of representation usually chosen for the molecular description of IDPs [13,14]. 

Due to the dynamic nature of IDPs, NMR spectroscopy is especially well-suited to probe their structure propensities and dynamics. While the lack of dispersion of proton resonances and severe signal-overlap causes problems in NMR of IDPs, intramolecular motions cause slower relaxation rates and allow the acquisition of spectra with narrow lines even for large proteins [5,15]. Advances in methodology, sample preparation techniques, labeling schemes, and NMR technology helped to overcome the problem of signal-overlap, thus enabling the study of more, larger IDPs by NMR spectroscopy [15]. However, intrinsically disordered regions are often part of large multi-domain proteins, connecting well-structured enzymatic domains and should be investigated in context with the superteriary structure of the protein [7]. In two recent studies, the supertertiary structure of the membrane associated guanylate kinase (MAGuK) PSD-95, a large scaffold protein with a number of disordered linkers connecting protein-binding domains, was resolved by employing single molecule FRET [16], and a combination of NMR and SAXS [17]. This underlines the importance of complementary approaches with low and high resolution techniques, with NMR spectroscopy typically being the method of choice for atomic resolution data. Several recently published articles review the structural characterization of IDPs by biophysical techniques, mostly with emphasis on NMR methods [5,18,19], or ensemble calculation [13,14], and one review exclusively discusses the progress in the field of ^13^C-direct detection [20]. Nevertheless, the emerging technique of in-cell NMR and its significance for the study of IDPs in a biological environment is not considered.

In this review, we will not only address NMR experiments and strategies that yield data which can be used for the generation of detailed structural models of IDPs, but we will also look at in-cell NMR where IDPs can be studied in a more physiological, crowded environment than in traditional in vitro experiments. Additionally, advances in ^13^C-direct detected experiments designed for the study of IDPs will be discussed. 

## 2. Resonance Assignment

**Figure 1:**
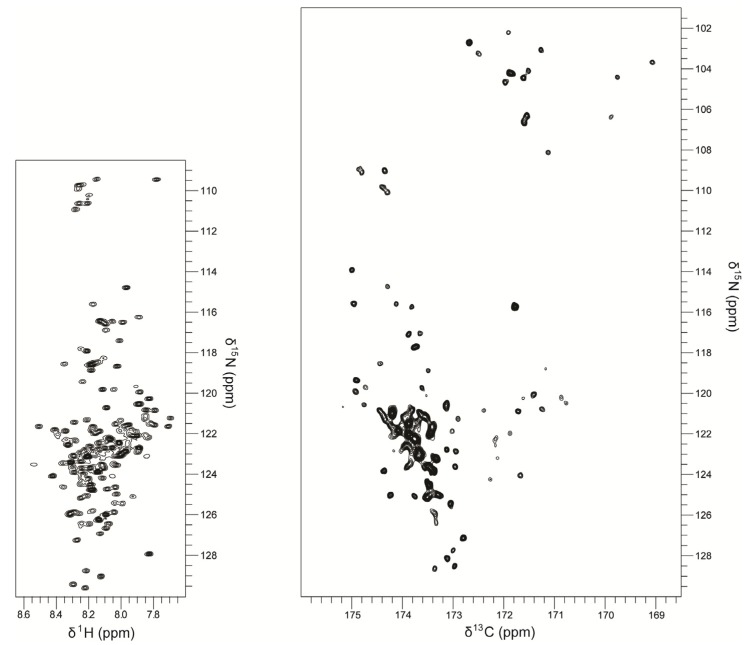
TROSY-^15^N-HSQC (left) and CON (right) spectra of the fully disordered protein ERD14. The comparison of both spectra clearly shows an improvement on the chemical shift dispersion by going from ^1^H- to ^13^C-detected experiments: While in the ^15^N-HSQC all the ^1^H frequencies are clustered within an area of 1 ppm (8.6–7.6 ppm region), ^13^C’ resonances are still distributed over the range of 7 ppm (176–169 ppm). *Acquisition of the spectra was done at 600 MHz Bruker spectrometer at 15 °C by using a CryoProbe TCI. Estimated protein concentration 50 μM (10 mM MES, pH 6.5).*

The first step to obtain structural information by NMR measurements is the assignment of resonances, which, in the case of IDPs, can be challenging due to extensive signal-overlap and exchange broadening of amide proton signals. For structured proteins, resonance assignment is done with a set of spectra, typically ^15^N, ^1^H resolved triple resonance experiments that contain sequential information by linking ^1^H_N_, ^13^C_α_, ^13^C_β_, C’ and ^15^N chemical shifts. However, for IDPs, the high shift degeneracy frequently demands a different approach. While the amide signal broadening can be overcome by H_α_-detection based approaches [21], signal-overlap is often handled by recording higher-dimensional spectra [22,23] or by employing ^13^C-detection [24]. The acquisition of 5D and 7D experiments, for instance, resolved signal-overlap sufficiently to allow automated assignment of tau [25]. The observation, that the resonances of ^13^C and ^15^N nuclei are more sensitive to primary structure and amino acid type, and therefore more dispersed, than ^1^H shifts, is generally exploited for the resonance assignment of IDPs [5,26]. ^13^C shift correlations are particularly well suited for resonance assignment of IDPs, and it has been shown that spectral resolution is greatly improved in ^1^H-^13^C′ spectra of an unfolded protein, compared to ^1^H-^15^N spectra (see Figure 1) [27]. This, together with the reduced influence of proton chemical exchange on line broadening, is further exploited in assignment strategies that utilize ^13^C-direct detection [24,28]. Several applications and ^13^C-direct-detection-based methods are discussed later in this review.

A different approach to avoid signal-overlap was used for the tau protein, which was divided into several small fragments that could be assigned individually. The obtained frequencies were then matched to the resonances of the full length IDP [29].

## 3. Structure and Dynamics Information from NMR Experiments

### 3.1. Structure Propensities—Local Structure

In IDPs, secondary structure is generally transient and confined to short segments that comprise only a small fraction of the total protein sequence. Structured elements can indicate posttranslational modification sites or the presence of binding motifs, and thus be of functional consequence. Propensities for alpha helices or extended strands are difficult to detect, and with techniques such as CD spectroscopy impossible to map onto the polypeptide sequence. The atomic resolution of NMR spectroscopy, however, allows the detection of such secondary structure propensities. Typically, chemical shifts and residual dipolar couplings (RDCs), with their high sensitivity to structure, are the preferred probes for secondary structure propensities, but J-couplings and, to some extent NOEs, have also been used in numerous studies. Examples include p27-KID [30], the p53 N-terminal transactivation domain [31], Sic1 [32], α-synuclein [33], and tau [29].

#### 3.1.1. Chemical Shifts

In structural studies of IDPs, chemical shifts are the most frequently used data that is obtained by NMR spectroscopy. The chemical shift is influenced by the local environment of a nucleus and therefore depends on the primary, secondary, and tertiary structure of the protein [34,35,36]. In solution, IDPs exist as interchanging conformers and the observed chemical shifts are consequently a population-weighted average of interconverting conformers over a time-scale up to ms. 

Secondary chemical shifts are determined as deviations of the experimentally determined chemical shift from random coil values. Generally, residues located in β-sheets have negative ^13^C_α_ and positive ^13^C_β_ secondary shifts, while amino acids in α-helices have positive ^13^C_α_ and negative ^13^C_β_ secondary shifts [36]. However, the need for a reference set of random coil chemical shifts implies the potential problem of introducing systematic errors if the referencing is incorrect. Several shift libraries and methods for analyzing experimental shifts have been developed from measurements of random coil peptides [37,38,39,40] or from analysis of protein databases [41]. In the case of IDPs, discrimination between the contribution of primary structure and secondary/tertiary structure to the chemical shift is of particular importance. Recent approaches to compile shift libraries and databases, such as CamCoil [34] or the ncIDP [35], consider the contribution of neighboring amino acids to the chemical shift. The use of a set of neighbor-corrected random coil shifts to detect transient secondary structure improves the reliability of structure propensity predictions [35]. Moreover, to interpret chemical shifts accurately, calibration with respect to temperature and pH is necessary [18,38,42]. The calculation of structure propensities from chemical shifts have been discussed in detail in two recently published reviews [43,44]. 

#### 3.1.2. Scalar Coupling

In addition to chemical shifts, scalar couplings can be used to obtain information about residual secondary structure. Based on the Karplus equations, backbone dihedral angles ϕ and Ψ can be derived from three-bond scalar couplings [45] and, due to the dependence of the angles on the conformation of the polypeptide backbone, secondary structure elements can be inferred. Positive deviations from couplings measured in random coil peptides, for instance, are typical for β-sheet-like structures, while negative deviations indicate α-helical propensity or polyproline helix type II [46]. Most studies use ϕ angles for conformational analysis as the Ψ angle is more difficult to obtain [47]. In practice, the coupling between H_N_ and H_α_ (^3^J_HNHα_) is usually used for the calculation of ϕ, although five other J-couplings can be exploited, as well [48]. However, most methods have been developed for measurement of ^3^J_HNHα_ in folded proteins as the coupling can be easily obtained from a pair of 2D spin-echo difference measurements in the form of HMQC and HSQC spectra, acquired with ^15^N labeled protein [49,50]. In ^1^H-^15^N correlation spectra of unstructured polypeptides, the extensive overlap of resonances frequently impedes extraction of a sufficient number of couplings for conformational analysis. With the increasing interest in IDPs, new strategies for the determination of ^3^J_HNHα_ coupling constants have been developed. Lendel and Damberg [51], for example, achieved improved resolution and reduced spectral overlap by removing the homonuclear coupling from the proton line-width in a 3D J-resolved ^1^H-^15^N correlation spectrum, which allowed them to determine an almost complete set of the coupling constants of the intrinsically disordered alpha-synuclein. Otten and colleagues [27], on the other hand, exploited the drastically improved signal resolution in ^1^H-^13^C′ correlation spectra of IDPs in an approach that uses a combination of HNCO triple resonance and HMQC-based spin-echo difference experiments [27]. But regardless of the chosen experimental approach, referencing to random coil values is necessary to detect transient secondary structure elements. Unfortunately, the analysis of scalar couplings suffers a similar risk of introducing systematic errors as chemical shift referencing does if the random coil values are not accurate. Overestimation of the propensity for secondary structure has been attributed to the lack of databases providing random-coil scalar coupling constants since only a small database with coupling constants of chemically denatured peptides is available [5,52].

### 3.2. Combined Long and Short Range Contact Information

#### 3.2.1. Residual Dipolar Coupling (RDC)

Another NMR method, frequently used in structural studies of IDPs, is residual dipolar coupling (RDC). The basis of this coupling effect is the behavior of every magnetic nucleus as a magnetic dipole that affects nearby nuclei. The method exploits the dependence of dipolar coupling on the angle the internuclear vector of two nuclei forms with the magnetic field vector [53]. The necessary partial alignment of the protein molecules in solution is possible with a number of media such as stressed polyacrylamide gels, lipid bicelles or filamentous bacteriophages [54]. In well-structured proteins, RDCs are used for structure refinement and determination of domain topology [55,56,57]. In conformationally heterogeneous samples, RDCs report on all orientations of internuclear vectors over an average of conformers interchanging on a timescale up to milliseconds. However, structured elements have an on average statistically preferred alignment to the magnetic field, so that N-H bond vectors are aligned in parallel or orthogonal depending on the type of secondary structure. The N-H RDC values for α-helical elements will be positive, while those for residues in extended structures will be negative, due to their orthogonally oriented N-H bond vectors [58]. To collect additional information, RDCs of a number of other intermolecular vectors can be measured to provide more accuracy in conformer descriptions [59]. The remarkable sensitivity for structured elements makes RDCs an excellent tool for probing structure propensities and conformational behavior of IDPs [54]. And, moreover, the analysis of RDCs of different bond vectors along the protein backbone allows the study of long-range order in structured proteins, and is sensitive to transient long-range order in IDPs [60,61]. 

The amount of information obtained from RDCs is difficult to incorporate in traditional representations of protein structures. In the recent years, different approaches have been developed where RDCs, together with chemical shifts, can be used to calculate ensembles of structural conformers. Mostly, a strategy that uses restrained molecular dynamics (MD) simulation, combining experimental restraints with a potential energy force field to introduce preferential conformational sampling, is employed to produce a structural ensemble [60,62,63]. Ensemble selection algorithms, such as ASTEROIDS or ENSEMBLE, select possible conformers according to their agreement with experimental data [60,64]. In a different approach, experimental data is not used to generate the model but to populate the calculated conformational space with regard to the experimental results [18]. It has been shown, that structural models of α-synuclein agree significantly better with experimental RDCs when long-range contacts are taken into account [60]. This demonstrates the importance of the combination of RDCs and long-range order information, derived from SAXS or PRE measurements, to avoid misinterpretation of RDCs in terms of local conformational behavior [60]. IDP ensemble calculation and description of IDP conformational behavior have been discussed in detail in recently published reviews [13,14,18,60].

Such an approach combining NMR and SAXS was used to determine the structure of the tumor suppressor p53 and its intrinsically disordered N-terminal transactivation domain. Together with electron microscopy data from previous studies, the structure of ternary complexes of p53 with DNA could be determined at residue resolution [31].

#### 3.2.2. Nuclear Overhauser Effect (NOE)

Structure determination of folded proteins by NMR spectroscopy is largely based on the distance information obtained from homonuclear proton NOEs. The NOE depends on the internuclear distance, with an upper limit of about 5 Å. Sequential and medium-range NOEs can usually be observed in IDPs, but long-range NOEs are rarely present in NOESY spectra. The absence of long-range NOEs, however, does not exclude the existence of contacts too short-lived to allow sufficient buildup of NOE signals. The fast dynamics of IDPs diminish NOE signals additionally, and if NOE signals are present, the high degeneracy of side chain chemical shifts makes unambiguous assignment of peaks difficult [11].

Nonetheless, NOEs have been employed in several structural studies of IDPs. For example, in the case of tau protein, short range NOEs have been used to confirm structure propensities inferred from chemical shifts [65]. Another study overcomes the difficulty of resolving and assigning H_N_-H_N_ NOE signals in NOESY-HSQC spectra with a selective labeling strategy that reduces the number of proton signals. This was combined with an increase in mixing times which allowed magnetization transfer up to 10 Å, and long and medium-range NOEs could be observed for the unfolded state of the drkN SH3 domain [66]. 

### 3.3. Long-Range Contacts

Due to the inherent flexibility of the polypeptide chain in IDPs, a number of transient long range contacts are expected. Several of these contacts, however, are not specific and arise from random proximity of regions of a random coil chain. Consequently, experimental data has to be evaluated and compared to statistical models taking the motional behavior of an idealized random coil ensemble into account [11]. In practice, models that predict experimental data in absence of specific, preferred contacts are calculated and then compared with measured data to extract true long range contacts. Information on long range contacts can be obtained from RDCs, occasionally from NOEs as described above, and from paramagnetic relaxation enhancements (PREs). In addition, techniques like SAXS, fluorescence correlation spectroscopy, and pulsed-field gradient (PFG) NMR are frequently used to determine the overall dimensions of an IDP.

#### 3.3.1. Pulsed-Field Gradient (PFG) NMR

Knowledge of the overall dimensions of an IDP allows conclusions about the existence of long range interactions within the molecule. Techniques able to measure the hydrodynamic radius (R_h_) or the radius of gyration (R_g_) of a protein can contribute valuable, complementary information. Pulsed-field gradient NMR allows the measurement of translational diffusion coefficients, which are used to determine formation of dimers or higher oligomers of folded proteins [67]. A number of different PFG experiments, also called diffusion ordered spectroscopy (DOSY), exists, which are in general based on the pulsed field gradient echo (PGSE) experiment. In the case of IDPs, PFG NMR is used to detect the presence of secondary structure elements or hydrophobic clusters that will reduce the hydrodynamic radius of the conformational ensemble [68]. Without additional data, however, it is not possible to identify the regions or residues involved. Another interesting application of diffusion measurements was recently described by Waudby *et al* who used pulsed-field gradients to distinguish between intracellular and extracellular protein [69]. The large difference in the diffusion coefficients allows the exclusive observation of intracellular protein by selectively dephasing resonances of extracellular protein [69].

#### 3.3.2. Paramagnetic Relaxation Enhancements (PREs)

**Figure 2:**
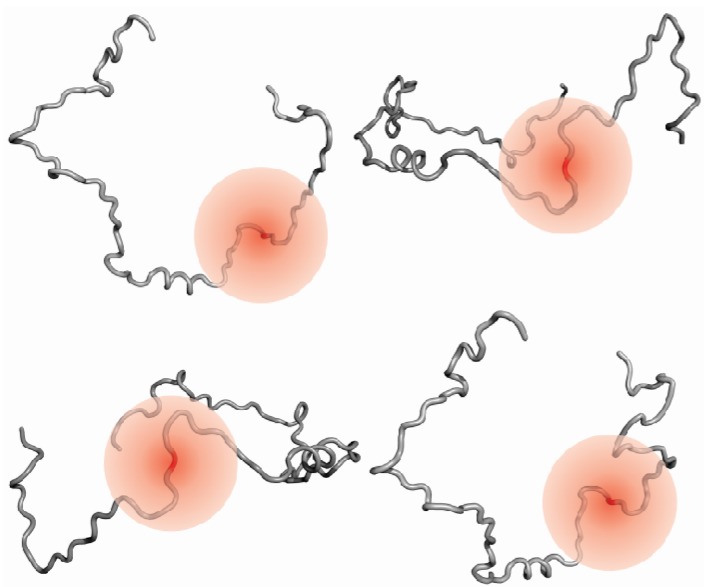
Paramagnetic probes for long-range contacts. If long-range contacts are present, residues sequentially distant from the tag will experience the PRE effect more frequently than would be expected from ideal random coil behavior. Different conformers will experience different PREs up to a range of 25Å as indicated by red spheres.

Utilized in the past to study chemically denatured, unfolded or partially folded proteins [70,71], paramagnetic relaxation enhancement (PRE) is probably the most frequently used NMR method to detect long range interactions of IDPs. The method is based on the dipolar interaction between nuclear spins and the spin of an unpaired electron of a paramagnetic component. Paramagnetic components induce increased relaxation rates and the degree of increase is described by the paramagnetic relaxation enhancement. The PRE depends on the correlation time and on the distance between the paramagnetic probe and the NMR active nucleus, thus providing distance information with an upper limit of 25Å [70]. The distance between a paramagnetic probe and an active nucleus can be calculated from the shortened relaxation time. In PRE studies of IDPs, a tag attached to a single amino acid is usually used as paramagnetic probe. In ideal random coils, the PRE experienced by nuclei of residues in the vicinity (10-15 residues away) of the residue carrying the tag will be strong and the corresponding resonances will be broadened. As the effect decreases with increasing distance from the paramagnetic probe, resonance signals of residues up or down stream in the sequence will experience less or no broadening. If long range contacts are present, nuclei of residues distant in the primary sequence can be spatially close to the tag and experience the PRE effect (see Figure 2) [11]. In literature, PREs are often extracted from a single pair of spectra, usually ^1^H-^15^N-HSQCs, acquired in the presence and absence of a paramagnetic probe. In this single time-point measurement the PRE relaxation rate is often underestimated, which can be easily overcome by using a two time-point measurement [72]. To obtain accurate PREs, it has been recommended to use transverse PRE rates as they are far less susceptible to internal motions and cross-relaxation than the longitudinal rates [73]. 

For PRE studies of IDPs, typically, a thiol-reactive nitroxide compound is conjugated to a single cysteine residue, which is introduced by site-directed mutagenesis at a desired position while other cysteines are removed from the protein. Different paramagnetic components can be introduced into the system by using different types of tags, native or engineered metal-binding sites, by incorporating non-natural amino acids, or by adding soluble paramagnetic molecules to the buffer [74,75]. The most frequently used paramagnetic probe for measuring PREs of IDPs, however, is the thiol-reactive methanethiosulfonate spin label (MTSL). The spin label has to be placed at carefully chosen positions to not influence or disturb structure formation, and it has to be considered that the mutations necessary for the introduction and removal of cysteine residues might affect the structure and dynamics of an IDP. 

Frequently, the spin label is introduced at several positions, to map all long range contacts present. In the case of the large tau protein, the wild-type protein and ten single-cysteine mutants were labeled with MTSL to reveal the existing long range contacts [29]. The same mutants were used in a recent study that utilized NMR, electron paramagnetic resonance, and SAXS to reveal detailed insights into the mechanism of tau aggregation inhibition and the structure and dynamics of soluble tau oligomers [76]. The tau mutants carrying the MTSL labels were further mutated to mimic multiple phosphorylated tau in order to study the effects of excessive phosphorylation on tau folding and aggregation as found in Alzheimer’s disease [77]. In the case of α-synuclein, PREs were used to map long-range interactions [78], and later as distance restraints in molecular dynamics simulations to determine its free energy landscape [33].

In a different approach, solvent PREs have been used to probe interactions of peptides and their hydrophobic sidechains with micelles acting as membrane mimetic. The water soluble paramagnetic component Gd(DTPA-BMA) provides a paramagnetic environment surrounding the micelles, and nuclei located in sidechains that are immersed in the micelle will experience a weaker PRE, given that the paramagnetic probe cannot enter the hydrophobic environment [79]. When Gd(DTPA-BMA) was titrated to samples containing micelles and the peptide hormone ghrelin in its active, posttranlationally octanoylated form, or in its inactive, non-octanoylated form, it could be shown that the active ghrelin adopted α-helical secondary structure upon binding to the micelles which was facilitated by its octanoyl moiety and an adjacent phenylalanine sidechain. This was not observed for the non-octanoylated ghrelin which did not interact with the micelles and remained intrinsically disordered, indicating a functional role of the membrane binding [80]. 

### 3.4. Information on Dynamics

IDPs are characteristically highly flexible, with different degrees of mobility attributed to different regions. Rigidity, or lack thereof, may be related to functional motifs. IDP flexibility has been linked to the affinity for target recognition, and the formation of dynamic complexes [3,32]. Moreover, inter-conversion rates between conformers might influence conformational selection in binding events, thus influencing function [81]. Characterization of dynamics, including backbone and sidechain flexibility, as well as inter-conversion rates, is therefore of great interest, and motivated the development of several techniques to explore the dynamic behavior of IDPs. Electron spin resonance (ESR) can provide backbone flexibility information, and fluorescence correlation spectroscopy (FCS) yields the timescale of conformational dynamics, if an appropriate tag is present [11]. NMR spectroscopy, on the other hand, can provide highly detailed dynamics information throughout the protein without the necessity of a tag. Mainly, ^15^N R_1_ and R_2_ relaxation measurements, as well as steady state ^1^H, ^15^N heteronuclear NOEs, are used for the characterization of backbone dynamics.

#### 3.4.1. ^15^N Relaxation

NMR relaxation studies are frequently used to obtain information on backbone dynamics, particularly the transverse ^15^N R_2_ CPMG rates are important indicators for protein backbone dynamics. They provide insights in backbone motions occurring on the ps to ns timescale and in conformational exchange processes on the µs to ms timescale [5,11]. Since a single global correlation time cannot describe IDPs, the model-free formalism used to describe the dynamics of globular proteins is not applicable for highly disordered proteins. Reduced-spectral-density mapping analysis represents a better alternative for the interpretation of relaxation data of IDPs [5,82]. Also, fast hydrogen exchange of unprotected amide protons with protons from the solvent can significantly affect measured R_2_, compromising its use in further analysis [83]. Instead, the ^15^N longitudinal relaxation rate in the rotating frame (R_1ρ_) or the cross-correlated relaxation rate (η) between ^15^H-^1^H dipolar and ^15^N chemical shift anisotropy interactions can be used [5,84]. Alternatively, the fast hydrogen exchange can be avoided by choosing acquisition conditions under which no such exchange exists, e.g. low temperatures or low pH, or the pulse sequence can be modified so that water saturation effects are removed [83]. Discrepancies between R_2_ and R_1ρ_ or η can also indicate conformational exchange on the µs-ms time scale, which can be inferred from the exchange-mediated transverse relaxation rate (R_ex_), which can be calculated either as the difference between R_2_ and R_1ρ_, or as the difference between R_2_ and η. This approach was used to show the slow backbone motion in the transient formation of a hairpin structure in the disordered amyloid β peptide [84].

#### 3.4.2. Heteronuclear NOEs

The heteronuclear NOE depends on the local effective correlation time and thus provides insights in the flexibility of different regions. ^1^H, ^15^N steady state NOE values are obtained by acquisition of one spectrum with and one spectrum without the use of 1H saturation applied before the start of the experiment. The heteronuclear NOEs are then simply obtained from the ratios of peak intensities in the saturated spectrum to those in the unsaturated spectrum [85]. In the case of flexible systems, ^1^H, ^15^N heteronuclear NOEs are sensitive reporters on the ps-ns time scale. They are frequently used in combination with ^15^N R_1_ and R_2_ relaxation measurements to provide a complete picture of the backbone dynamics of an IDP. ^1^H, ^15^N heteronuclear NOEs were used together with chemical shifts to study nascent structural and dynamic features of p27-KID, and elucidate its folding-on-binding mechanism critical for its cell-cycle regulatory function [30].

#### 3.4.3. Relaxation Dispersion

More recently, relaxation dispersion data has been used for the study IDP binding mechanisms, as well as for the extraction of the kinetics of exchange processes of transiently formed, low populated folding intermediates [86,87]. These metastable intermediates can generally not be detected by NMR as they are transiently formed and weakly populated. However, the intermediate conformers contribute to line broadening of visible peaks of other conformations. If the exchange occurs on the millisecond timescale, the line broadening can be quantified by recording the decay of transverse magnetization (R_2_) as a function of the strength of an applied radiofrequency field (ν_CPMG_) [88]. Recent progress in relaxation dispersion NMR allows the extraction of kinetics, thermodynamics and chemical shifts of these metastable states [89]. Thus, disordered regions in intermediate states can be detected as was the case for the C-terminus of the FF domain of HYPA/FBP11 [86,89]. In another study, relaxation dispersion was used in combination with NMR titrations to elucidate the binding mechanism of the intrinsically disordered KID domain of CREB to the transcriptional coactivator CBP [87]. It could be shown that, initially formed, transient encounter complexes evolve to the fully bound state which is stabilized mainly by hydrophobic contacts [87].

## 4. ^13^C -Detected NMR for IDP Studies

### 4.1. Introduction: How ^13^C -Detection Can Help

^1^H-detected experiments have been routinely applied to study protein structures and dynamics due to their higher sensitivity. Protons are characterized by their large gyromagnetic ratio, compared to other heteronuclei (^13^C, ^15^N), resulting in an enhanced sensitivity during the acquisition [90]. Nitrogen and carbon shifts can be determined from correlation signals, making ^1^H-detected experiments a convenient tool for the study of proteins [91].

Nonetheless, ^1^H is not the most suitable nucleus for IDP studies. Peptide backbones of IDPs have high solvent-exposure, which leads to an increase of the amide proton exchange rates that can cause broadening of the signal [28]. Dipolar interactions may also contribute to line-broadening and the lack of a unique 3D structure drastically reduces the ^1^H dispersion, while in some cases ^13^C-nuclei shifts are still well-dispersed (Figure 1) [90]. Also, due to the fast relaxation of protons line broadening is a considerable problem when applying ^1^H-detected experiments to large macromolecules. However, this problem can be solved by taking advantage of the slower relaxation of ^13^C. Additionally, extra information about backbone and side-chains of all amino acid types, including the highly abundant prolines in IDPs, can be extracted by exploiting the different ^13^C types (C’, C^α^, C^aliph^, C^aro^) in ^13^C-detected experiments [90].

Thanks to the increase in spectrometer sensitivity, ^13^C-detection is starting to be frequently applied on biological macromolecules. Since 1999, cryogenically cooled probes (CryoProbes or Cold probes) have been implemented in spectrometers helping in cases where protein concentration is too low, experiment time has to be reduced or an increment of signal-to-noise ratio is needed [92,93]. More recently, optimized CryoProbes for ^13^C detection have been developed [94]. Also, the development of more powerful NMR spectrometers in conjunction with methodological and technical advances resulted in an increased sensitivity. For the study of IDPs, ^13^C-detection NMR spectroscopy opens many possibilities, and many others still have to be explored. Nonetheless, ^13^C-detected experiments should be considered as a complementary tool to ^1^H-detection for challenging systems such as unfolded proteins, very large proteins or parts of proteins affected by high proton exchange processes. However, the applications can be limited by the rather high-concentrated protein samples that are required for ^13^C-detected NMR experiments.

Several strategies based on a variety of ^13^C-detected experiments have been developed to obtain structural and dynamics information of IDPs and other systems suffering from severe signal overlap in ^1^H-detected experiments.

### 4.2. Sequential Protein Backbone Assignment with ^13^C-detected Experiments

The strategy for backbone assignment using ^13^C-detected experiments follows the same principles as ^1^H-detection, with differing starting and ending points of the experiment. In case of ^13^C-detected experiments, the magnetization starts from ^13^C nuclei (C^α^ or C’) and is finally detected at the same or another ^13^C, depending on the approach used, “out-and-back” or “out-and-stay”, respectively. However, in many cases, higher resolution within a shorter experimental time is obtained with the last approach [94].

Improvement of chemical shift dispersion can be achieved by exploiting correlations between inter-residue nuclei [28]. However, the gain in resolution by using heteronuclei and inter-residue correlations strongly depends on the homonuclear ^13^C decoupling. Many methods are available in literature, although in most cases, the IPAP and S2E filters achieve higher sensitivity than other methods of J-coupling suppression [28,95,96].

**Figure 3:**
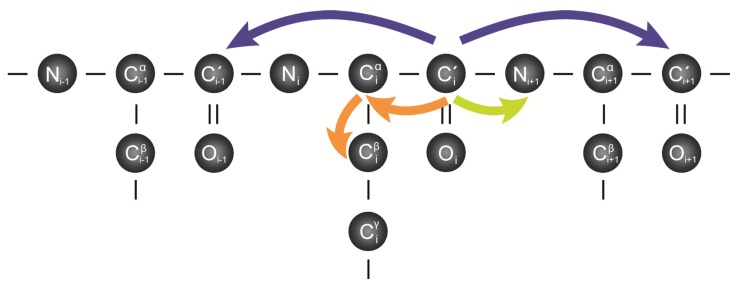
Correlations observed in CON based experiments. Schematic representation of the detected correlations by using the following ^13^C-direct detection NMR experiment type: 3D COCON experiment (purple), 3D CBCACON (orange) and 2D CON (green).

Complete backbone assignment of an IDP can be accomplished by applying different types of experiments. Generally, ^13^C-^15^N labeled samples are used and in many described cases a full set of ^1^H-detected experiments for backbone assignment was already available. For instance, three experiment types were employed to reassign α-synuclein by only using ^13^C-detected experiments (7): 3D COCON, 3D CBCACON and 2D CON. The 3D COCON experiment connects the backbone nitrogen (N_i+1_) with the attached carbonyl carbon (C’_i_) and with the previous and the following carbonyl carbons (C’_i-1_, C’_i+1_) in the sequence. And the 3D CBCACON yields the correlation of backbone nitrogen (N_i+1_) with the attached carbonyl carbon (C’_i_) and intraresidue C^α^_i_ and C^ß^_i_ (Figure 3) [24]. 

In a different approach, the 22kDa IDP securin was assigned from a suite of experiments utilizing ^13^C-detection, but, to improve sensitivity, with ^1^H polarization as starting source. This resulted in an increased signal-to-noise ratio, and increased resolution due to the larger heteronuclei chemical shift dispersion [97]. Three 3D experiments were used: *3D (H)CBCACON*, *3D (H) CBCANCO*, *3D (H)NCANCO.* The combination of a *3D (H)CBCACON* and a *3D (H)CBCANCO* experiment allows an extra correlation with the inter-residue C^α^_i+1_ and C^ß^_i+1_. *3D (H)NCANCO* correlates the backbone nitrogen (N_i+1_) with the neighboring carbonyl carbon (C’_i_) of the backbone with three nitrogens (N_i_, N_i+1_, N_i+2_). Nevertheless, the use of other experiments both ^1^H- and ^13^C-detected experiments such as CANCO, CACON, inter-CAN (see Table 1) can be really helpful [97].

### 4.3. ^13^C-detected Amino-Acid-Selective (CAS) NMR Experiments

In this innovative experimental design, amino-acid-selective editing blocks are introduced in the CACON- and CANCO-type sequences. Correlation between a specific type or group of amino-acids is shown and the necessary inter- and intra-residue connectivities are provided by the standard CACON and CANCO experiments. Two main advantages arise from this method: firstly, the resulting spectrum is comparable to one obtained by amino-acid-selective labeling, although with only one sample being sufficient, and secondly, the extreme simplification of the spectra, reducing the strong amino-acid-type overlap characteristic of IDPs [98].

### 4.4. Probing Arginine Side-Chains

Distinct ^13^C-detected experiments have been developed to use arginine side-chains as a probe for studying protein function and dynamics. The large, charged arginine side-chains are almost uncoupled from the backbone, probing a different environment from that of the backbone or the hydrophobic side-chains. They, therefore, provide valuable information about protein interactions and molecular motions [99]. 

Previous approaches to probe arginine and lysine side-chains were based on aliphatic proton detection, preventing line-broadening by rapid amide proton exchange [100]. Furthermore, the novel pulse sequence based on the ^13^C_ζ_-^15^Nε correlation (^13^C_ζ_-^15^Nε HSQC) allows the characterization of arginine side-chains of larger molecules at physiological or higher pH [99]. The recently developed method was, at this point, only used for structured proteins but will provide an interesting tool for probing interactions of IDPs which frequently utilize electrostatic effects in molecular recognition [101].

### 4.5. Studying Protein Dynamics Based on ^13^C-Detected Experiments

#### 4.5.1. ^13^C-Detected ^15^N NMR Spin Relaxation

Relaxation studies are essential to understand the conformational dynamics of IDPs (ensemble description, disorder-to-order transition, etc). However, the low dispersion of ^1^H-detected spectra restricts NMR relaxation experiments to a limited number of proteins. Recently, ^15^N T_1_ and T_2_ relaxation measurements have been done by using an approach based on ^1^H-start CON experiments, CON(T_1_)-IPAP and CON(T_2_)-IPAP [102]. This novel pulse sequence allowed, for the first time, to experimentally confirm that in the FCP1-RAP74 complex, described in literature as a “fuzzy” complex [4], FCP1 remains in a highly disordered state even upon binding [102].

#### 4.5.2. RDCs and J Couplings by ^13^C-Detected NMR Spectroscopy

Coupling constants can be also measured by ^13^C direct-detected methods avoiding the ^1^H NMR line broadening in specific systems such as large, paramagnetic or flexible proteins. Protonless ^13^C-detected experiments were designed for measuring *J_C_^α^_C’_*, *J_C_^α^_C_^ß^*, *J_H_^α^_C_^α^* and *J_HNN_* couplings, the last two being necessary to reintroduce HC coupling. This collection of coupling constants permits determination of peptide backbone orientation and the angles around C^α^ nuclei [103]. A more recent publication [104], describes a method to measure C^α^-C’, C’-N and C^α^-H^α^ RDCs by using the CON-type experiment, and additionally, the relaxation times (T_1_ and T_2_) of N and C’ (see table 1). The feasibility of the method was demonstrated with the folded ubiquitin and might prove useful to obtain dynamics information of IDPs with strong signal-overlap in ^1^H detected spectra. 

**Table 1:** Set of experiments described in the ^13^C-detected NMR methodology. Only relevant correlations for the experimental aim are mentioned (NA, Non Available; IPAP, In-Phase Anti-Phase; SE-DIPAP, Storage Element Double In-Phase Anti-Phase; CPD, Composite Pulse Decoupling).

Experiment Type	Use/Aim	Correlations Observed	J-Coupling Supression Used	[Protein] Used	Reference
3D COCON	Backbone assignment	C'_i_-C'_i_-N_i+1_, C'_i-1_,-C'_i_-N_i+1,_ C'_i+1_ -C'_i_ -N_i+1_	IPAP	NA	[24]
3D CBCACON	Backbone assignment	C^α^_i_-C'_i_-N_i+1_, C^ß^_i_-C'_i_-N_i+1_	IPAP	NA	[24]
2D CON	Backbone assignment	C'_i_-N_i+1_	IPAP, SE-DIPAP	NA	[24]
3D (H)CBCACON	Backbone assignment	C^α^_i-1_-C'_i-1_-N_i_, C^ß^_i-1_-C'_i-1_-N_i_	IPAP	0.7 mM	[97]
3D (H)CBCANCO	Backbone assignment	C^α^_i_-C'_i-1_-N_i_, C^ß^_i_-C'_i-1_-N_i_, C^ß^_i_-C'_i_-N_i+1_, C^α^_i_-C'_i_-N_i+1_	IPAP	0.7 mM	[97]
3D (H)NCANCO	Backbone assignment	N_i_-N_i_-C'_i-1_, N_i+1_-N_i_-C_i-1_, N_i-1_-N_i_-C'_i-1_	IPAP	0.7 mM	[97]
3D CANCO	Backbone assignment	Cαi-C'i-Ni+1, C^α^_i+1_-C'_i_-N_i+1 _	IPAP	1–1.8 mM	[98]
3D CACON	Backbone assignment	C^α^_i_-C'_i_-N_i+1_	IPAP	1–1.8 mM	[98]
^13^C_ζ_-^15^Nε HSQC	Probing Arg side-chains	C^ζ^_i_-N^ε^_i_	IPAP, CPD	0.8–2.5 mM	[99]
2D CON-type	Relaxation measurements RDC’s	C'_i_-N_i+1_	IPAP	1 mM	[102,104]
			IPAP	0.5 mM	

#### 4.5.3. Amide Proton Solvent Exchange

As last example, another CON-type (HNflipX-CON) experiment has been used for determining exchange processes of backbone amide protons with the solvent. This method allows the establishment of local flexibility by monitoring the solvent accessibility. In addition, Bertini and colleagues [105] proposed other possible experiments to measure different relevant key observables for protein dynamics.

## 5. In-Cell NMR Spectroscopy

The lack of well-defined structure, as well as the inherent high flexibility of IDPs in in vitro experiments, raises the question of their dynamic behavior in cellular environments which are, on the molecular level, more crowded and viscous than buffer systems. A disordered protein freely diffusing in a test tube does not necessarily behave like a protein embedded in a cell matrix, exposed to other macromolecules, metabolites, membranes and local pH changes. Shifts within a given population of interconverting structures, an increase in structure and decrease in flexibility can occur, and binding events can be affected from a kinetic point of view. A number of agents such as PEG, Ficoll or dextrans that mimic the crowding effect can be used in vitro studies. However, no significant conformational changes were detected when the effects of macromolecular crowding on several IDPs and IDRs were investigated by NMR spectroscopy [106,107,108], small angle neutron scattering [109], or CD and fluorescence spectroscopy [110]. 

In a living cell with an active metabolism, the post-translational modifications and interactions with other proteins and cellular components have to be taken into consideration in addition to the crowding by other, non-interacting macromolecules. In in-cell NMR spectroscopy, proteins can be studied with atomic resolution in intact cellular systems, thus allowing the observation of regulatory events such as phosphorylations in real-time [111]. IDPs are, due to their correlation times in the range of ns to ps, ideally suited for in-cell NMR studies and show little to no peak broadening or signal loss under crowding conditions [107]. Recent progresses in in-cell NMR methodology allow the monitoring of post-translational modifications [111,112], protein-protein interaction [113], global protein motion [114], and even structure calculation from in-cell spectra [115]. The most common use, however, is to probe binding interactions and structural modifications directly from proton nitrogen correlation spectra. 

Critical for in-cell data acquisition are the preparation of samples by introducing an isotope labeled protein into live cells and the survival of the cells during the measurement [116]. Several methods for protein delivery into prokaryotic and eukaryotic cells have been developed and a number of cells have been used in in-cell NMR studies (Table 2).

**Table 2:** Types of cells, delivery methods, proteins, and experiments used in in-cell NMR. MerA: bacterial putative mercuric reductase; GB1: B1 domain of streptococcal protein G; SOD1: Superoxide Dismutase 1; c-Src: proto-oncogene c-Src or non-receptor tyrosine kinase; Atx1: Metal homeostasis factor Atx1; FKBP: FK506 binding protein; FRB: rapamycin binding domain of mTor; TTHA1718: Heavy metal binding protein from *Thermus thermophiles* HB8; ProtL: immunoglobulin G binding domain of protein L.

Cell line	Delivery	Protein studied	Studies	References
HeLa (human) COS7 (*Chlorocebus*)	Peptide tag	Ub, FKBP12, GB1	Protein–drug interaction, enzymatic cleavage, H/D-exchange	[117]
293-F (human)	Pore-forming toxin	thymosin ß4	N-terminal acetylation	[118]
HeLa cells		α-synuclein	Conformation	[119]
*Xenopus laevis*	Microinjection	Viral SV40 large T antigen regulatory region	Protein phosphorylation	[120]
		Tau protein	Interaction with microtubules, protein phosphorylation	[112]

		GB1	Macromolecular crowding	[121]
Human embryonic kidney (HEK293T)	Overexpression	Human SOD1	Monitor folding	[122]
Insect cells	Overexpression	G B1	Chemical shift assignment	[123]
*Pichia pastoris* (budding yeast)	Overexpression	Ubiquitin	Assessment of critical parameters for the cell type, metabolism effect	[124]

*Escherichia coli*	Overexpression	NmerA	Proof of concept	[125]
		Atox1	Cis-platin transport	[126]
		Ubiquitin	Protein-protein interaction	[127]
		FKBP-FRB	Drug screening	[117]
		Human SOD1	Monitor folding	[128]
		TTHA1718	Structure determination	[115]
		ProtL	Folding	[129]
		Chymiotrypsin inhibitor 2 (CI2)	Correlation spectra	[107]
		α-synuclein	^13^C-direct detection	[130]

### 5.1. Protein Delivery Methods for In-Cell NMR

Depending on cell type and availability of technological tools, proteins can be introduced into cells either by over-expressing the target protein using an inducible plasmid, or by delivering it from outside. Induced over-expression of the protein of interest allows control over protein concentration via the promoter, and has the advantage of avoiding any transfer or extracellular manipulation steps. Over-expression has been used in prokaryotic and eukaryotic cells, including insect cells and human cell lines [116,122,123]. Only recently, Banci *et al.* [122] described the complete maturation and folding process of human superoxide dismutase 1 directly expressed in HEK293T cells by in-cell NMR. 

Cell penetrating peptides, reversible pore-formation or microinjection, on the other hand, can be used to deliver a recombinantly expressed, labeled protein into the desired cellular environment [116,118]. Cleavable tags of a cell-penetrating peptide derived from the Tat protein of HIV-1 have been used to study different proteins in HeLa cells. The formation of complexes of FKBP12 with immunosuppressants in HeLa cells could be observed with this delivery technique [117]. In a similar approach the disordered α synuclein was delivered to HeLa cells by linking it to the peptide tag via an oxidative, disulfide-coupling reaction [131].

In a study by Ogino *et al.* [118], isotope labeled thymosin β4, an intrinsically disordered actin-sequestering protein, was introduced in human embryonic kidney cells by utilizing the pore forming properties of streptolysin O. The same technique has been used to transfer α synuclein into HeLa cells [119].

Microinjection is limited to large, easily manipulated cells, such as *X. laevis* oocytes, but offers the advantage of exact protein concentration control. Transfer of proteins expressed in bacterial systems into *X. laevis* oocytes is frequently utilized for post-translational modification studies [111,112], due to the lack of post-translational modifications in E. coli cells.

### 5.2. Monitoring Post-Translational Modifications with In-Cell NMR

The function of IDPs is frequently regulated by post-translational modifications (PTM) and in multi-domain proteins, IDRs are preferred sites of modifications such as phosphorylation, acetylation or methylation [8]. In-cell NMR is especially well-suited to study these modifications as they can be carried out directly in vivo by the cell’s own machinery [111,120,132]. Most post-translational modifications such as serine and threonine phosphorylation, or lysine acetylation or methylation are easy to detect in simple 2D ^1^H–^15^N correlation experiments due the large shift changes in the amide proton signals [132,133,134].

Novel phosphorylation events could be detected when tau protein was studied in *Xenopus* oocytes [112], and in a recent in-cell study, the effects of *X. laevis* oocyte kinases and phosphatases on a disordered domain of c-Src were studied in real-time [111]. Again in microinjected *Xenopus* oocytes, Selenko *et al.* [120] uncovered the mechanism of a stepwise, consecutive phosphorylation of two neighboring casein kinase substrate sites located in the disordered SV4 large T antigen regulatory region.

N-terminal acetylation, a modification frequently occurring in eukaryotic cells, was shown for the IDP thymosin β4 in human 293-F cells [118].

### 5.3. Protein Interactions

In-cell interaction studies with endogenous interaction partners are in most cases hampered by too low cellular concentrations of most proteins. Nonetheless, the association of tau with microtubules, which have a concentration of about 20 µM in *Xenopus* oocytes has been shown [112]. In titration studies, cell viability is difficult to maintain. In bacterial cells, the STINT-NMR method can be used to study protein-protein interactions. In this technique, the interactions of two proteins, which are sequentially expressed within a single bacterial cell in a time-controlled manner, can be monitored in ^15^N-HSQC spectra [127]. This method, however, has not been applied to IDPs yet. 

### 5.4. ^13^C direct Detection In-Cell

The feasibility of ^13^C direct detected experiments *in-cell* was demonstrated by Bertini and collaborators [130] on folded Atx1 protein and α-synuclein inside *E.coli*. Typically, the CON experiments showed better dispersion for α-synuclein signals, and more resonances can be assigned to a larger number of residues compared to an equivalent experiment of Atx1 [130]. 

## 6. Conclusion and Outlook

Because of the lack of stable secondary or tertiary structure and their high flexibility, the conformational state of IDPs is described by extensive ensembles derived from various experimental data. Ensembles are simplified presentations of large datasets, frequently including data from additional biophysical techniques, such as SAXS, EPR or single molecule FRET. The need for combination of different methods to provide a comprehensive structural framework for understanding the functional biological context has become evident in recent structural studies, such as the ensemble characterization of the measles virus where a joint approach of NMR, small angle scattering (SAS) and electron microscopy was used [135]. Particularly for the structural characterization of intrinsically disordered regions that are often part of large multi-domain proteins, utilization of low and high resolution methods is indispensable. Recent advances in single molecule FRET methodology allowed the observation of IDRs in the context of the full length protein [16]. In our lab, we currently study the supertertiary structure of the large multi-domain co-transcription factors CBP and p300 by combining X-ray crystallography and NMR data of structured and disordered domains with high speed AFM, single molecule FRET and cryo-EM.

The effect of molecular crowding, as present in the cell, on the structure and dynamic behavior of IDPs has been frequently debated. Although physiological buffers can resemble the biological conditions in the cell if a crowding agent is added, it is desirable to obtain structural and dynamics information directly from proteins in cells. For functional studies and analyses of interaction regulation, it can be of particular interest to observe post-translational modifications such as phosphorylation patterns. The recent progress in in-cell NMR allows the study of IDPs in cellular environment and phosphorylations, acetylations, and methylations can be detected. Approaches that additionally utilize the advantages of ^13^C-direct detection can contribute considerably to understanding structure and function of IDPs in cellular environment.

NMR spectroscopy is a powerful tool for structural and dynamics studies of IDPs in vitro and in-cell. Nonetheless, to obtain a complete picture of the complicated conformational ensembles, and of IDP function regulation by changing the conformational equilibrium in these ensembles through post-translational modifications, complementary approaches with other biophysical techniques are necessary.
